# Privacy threats versus trust: a behavioral decision approach to social media disclosure intention

**DOI:** 10.3389/fpsyg.2025.1609012

**Published:** 2025-09-25

**Authors:** Luhui Zhu, Changfu Zhang, Bahiyah Omar, Fei Qi, Hongchao Ji

**Affiliations:** ^1^School of Communication, Universiti Sains Malaysia, Penang, Malaysia; ^2^School of Engineering Economics, Henan Finance University, Zhengzhou, China; ^3^School of Literature, Guizhou University of Finance and Economics, Guiyang, China; ^4^Hinterland Development Research Institute, Henan University, Zhengzhou, China

**Keywords:** social media disclosure intention, privacy invasion experiences, privacy concerns, privacy fatigue, trust in social media, behavioral decision theory

## Abstract

**Introduction:**

As social media becomes a central platform for self-expression and communication, users are increasingly faced with the dilemma of disclosing personal information while managing privacy risks. This study explores how privacy-related factors, namely privacy invasion experiences, privacy fatigue, and privacy concerns, are associated with users’ intention to disclose on social media, with trust in social media serving as a mediating variable. Integrating behavioral decision theory and trust theory, the study aims to uncover the psychological mechanisms driving social media disclosure intention in a digital context.

**Methods:**

A quantitative survey was conducted with 787 participants to examine the proposed relationships. PLS-SEM was employed to test the hypothesized paths and mediating effects within the theoretical framework.

**Results:**

The results demonstrate that trust in social media is the most important predictor of social media disclosure intention, exceeding the negative impact of privacy-related factors. All three privacy-related variables negatively influence users’ intention to disclose, with privacy concerns showing the strongest negative effect. Trust in social media mediates the relationship between privacy invasion experiences and privacy concerns and social media disclosure intention. Furthermore, we found no significant relationship between privacy fatigue and trust in social media.

**Discussion:**

The study extends existing theory by applying behavioral decision theory to the digital privacy domain and underscores the importance of trust in social media as a psychological bridge between privacy threats and social media disclosure intention.

## 1 Introduction

Social media disclosure has become an indispensable aspect of users’ daily lives, serving to enhance social connections, facilitate self-expression, and promote the exchange of information (Chen et al., 2025). By sharing personal experiences, thoughts, and emotions online, individuals strengthen interpersonal relationships, obtain social support, and engage with broader communities. However, despite these significant benefits, such disclosure also exposes users to potential privacy risks, including unauthorized data access and identity theft (Chaudhuri et al., 2023). As a result, privacy threats have emerged as critical negative factors influencing users’ intention to disclose personal information in online environments.

As an emotional response after experiencing privacy issues, privacy concerns have received extensive attention, yet scholarly understanding of its role in shaping disclosure and trust remains inconsistent across studies (Maseeh et al., 2021). Additionally, individuals may feel exhaustion, frustration, or helplessness due to the constant need to manage and protect their personal information in the digital age, this phenomenon is recognized as privacy fatigue (Tang et al., 2021). Privacy fatigue has emerged as a critical factor influencing users’ attitudes and behaviors in digital environments, and many studies have focused on its causes and its impact on social media usage behavior (Choi et al., 2018; Van Der Schyff et al., 2023; Wang W. et al., 2025). However, empirical studies on its role in the trust-disclosure dynamic remains limited (Chen et al., 2023). Given the increasing public awareness of privacy issues and the growing severity of privacy invasions, it is crucial to explore how users’ privacy invasion experiences affect their privacy-related emotional and attitudinal factors and further influence their trust in social media, which finally affects their intention to disclose on social media.

Despite escalating privacy threats, users continue to disclose information online even when they express privacy concerns or have experienced privacy invasions. Scholars have made great efforts to explain this puzzle. A potential explanation for this paradox is the role of trust in online platforms (Zhang et al., 2025). Trust has long been recognized as a fundamental prerequisite for social and economic transactions, playing a central role in commerce, cooperation, and the stability of society (Zhang et al., 2023). Trust becomes even more important in the digital age, especially in the context of social media where users frequently engage in self-disclosure in their daily lives. Previous research has established that trust in social media influences users’ willingness to disclose personal information online (Apuke and Omar, 2021; Ruangkanjanases et al., 2022; Wang J. et al., 2025), however, despite its recognized importance, there has been insufficient systematic investigation into how users’ personal experiences of privacy invasion, as well as their perceptions and emotional responses to privacy issues, jointly impact social media trust and further influence disclosure intention on social media.

In applied research on social media disclosure, scholars have drawn on multiple theoretical perspectives (Jhawar et al., 2024). However, behavioral decision theory is particularly well-suited for examining disclosure behavior, because it explicitly accounts for the cognitive and psychological biases that affect individual decision-making. Behavioral decision theory posits that human behavior is not purely rational but instead influenced by anchoring effects, cognitive biases, and loss aversion (Gilovich et al., 2002; Tversky and Kahneman, 1974). Building on this foundation, our study conceptualizes privacy invasion experiences as anchoring events, privacy fatigue as a manifestation of cognitive bias, and privacy concerns as an expression of loss aversion. Integrating these constructs into trust theory enables us to investigate how users’ psychological responses to privacy-related threats influence their trust in social media and subsequent disclosure intention. Specifically, we explore the relationship between trust in social media and disclosure intention. Furthermore, we explore how privacy invasion experiences, privacy fatigue, and privacy concerns influence users’ trust in social media and disclosure intention, as well as how privacy invasion experiences influence privacy fatigue and privacy concerns. Additionally, age and gender are accounted as control variables in our study.

To explore these relationships, we develop a research model grounded in behavioral decision theory and trust theory. Our study employs a quantitative approach using PLS-SEM to analyze survey data collected from 787 social media users in China. By integrating privacy behavioral decision factors as independent variables and trust as a mediating role, this research provides a comprehensive perspective on how privacy-related decision-making factors interact and collectively impact users’ intention to disclose personal information through the mechanism of trust.

## 2 Literature review

### 2.1 Social media disclosure intention

Social media disclosure intention refers to individuals’ behavioral tendency to reveal personal information on social media platforms. It is a key predictor of actual disclosure behavior online, which has received sustained attention in privacy and communication research. According to Norberg et al. (2007), disclosure intention reflects the motivational component of the privacy calculus, revealing how users evaluate the trade-off between perceived benefits and perceived risks. More recently, scholars have emphasized its role in shaping digital identity and online interaction patterns (Rodríguez-Priego et al., 2023).

A number of factors have been identified as influencing social media disclosure intention. Trust in social media, for example, plays a pivotal role in reducing perceived risks (Rodríguez-Priego et al., 2023) and encouraging information sharing (Omar et al., 2024). When users believe that a platform is secure, reliable, and respects their privacy, they are more likely to engage in disclosure (Nemec Zlatolas et al., 2019). Additionally, individual differences, such as personality traits and risk tolerance, further moderate disclosure intention. Studies have shown that users with a higher propensity for risk-taking or with an extroverted personality may exhibit greater disclosure intentions, whereas those with more conservative traits might be more reserved (Chen et al., 2016). The literature on social media disclosure intention underscores the importance of context factors as well. For instance, Bansal et al. (2016) found that the specific nature of the information being shared and the perceived sensitivity of the context can significantly affect users’ decisions to disclose.

However, there is still an inconsistent understanding of the negative role of privacy concerns on social media disclosure (Cain and Imre, 2021). One stream considers this negative effect to be significant [e.g., Lutz et al. (2018), Gruzd and Hernández-García (2018)], while the other considers it to be insignificant [e.g., Barth et al. (2019), Hughes-Roberts (2013)]. Moreover, its interplay with past experiences with privacy invasion and privacy fatigue in influencing social media disclosure intention needs further clarification.

### 2.2 Privacy invasion experiences, privacy fatigue, privacy concerns

Behavioral decision theory is a framework for understanding how individuals make decisions under uncertainty and in complex situations (Einhorn and Hogarth, 1981). This theory shows that individual behavioral intention is influenced by a variety of factors, including anchoring, heuristics, and loss aversion. The anchoring effect suggests that individuals rely heavily on initial information when making decisions, and prior encounters with privacy breaches can act as a powerful reference point in shaping subsequent perceptions (Furnham and Boo, 2011). Heuristics are simplified decision-making rules that people use to save cognitive resources and time when facing complex problems. Heuristics help people make decisions quickly, but they can also easily lead to cognitive biases (Dale, 2015). Loss aversion refers to the emotional response that people are more sensitive to losses than to gains of equal value (Novemsky and Kahneman, 2005); that is, people are more willing to avoid losses rather than pursue equal gains. Drawing on insights from psychology and cognitive science, scholars posit that decision-making is influenced by cognitive biases, emotional responses, and past experiences (Beldhuis et al., 2021; Naqvi et al., 2006).

Specifically, past experiences always play a pivotal role in influencing future decisions. Privacy invasion experiences refer to individuals’ past encounters with unauthorized access, misuse, or exposure of their personal information in digital environments (Huang and Leu, 2020). When users experience unauthorized access or misuse of their personal data, these events become salient anchors that influence how they evaluate the risks associated with disclosing further information on social media. Drawing on anchoring theory, previous encounters with privacy invasions function as cognitive anchors, influencing users’ perceived vulnerability and decision-making patterns in similar contexts (Acquisti et al., 2015). Recent research confirms that individuals who have experienced privacy invasions are more likely to exhibit heightened risk awareness and lower platform trust (Chen et al., 2024). These anchoring effects create a cognitive frame through which users assess the safety and reliability of disclosing personal information on social media.

Privacy fatigue is conceptually defined as a psychological state of weariness and resignation that arises when individuals feel overwhelmed by the effort required to manage and protect their personal data online (Chen et al., 2023; Choi et al., 2018). This condition is often triggered by increasing data breaches, insufficient control over personal information, and frequent exposure to complex privacy decisions (Acquisti et al., 2015; Smith et al., 2011). As users encounter frequent privacy invasions, they are often required to engage in cognitively demanding tasks, such as navigating dull privacy policies or configuring complex security settings. For many individuals, protecting privacy becomes an exhausting task. The complexity of safeguarding personal information exacerbates feelings of helplessness, inability and exhaustion, ultimately leading to privacy fatigue. This condition reflects heuristic-driven cognitive biases. When individuals face mental overload, they tend to simplify decision-making processes, often adopting avoidance or withdrawal strategies in relation to social media disclosure (Van Der Schyff et al., 2023). Thus, privacy fatigue is not merely a passive response to privacy invasions; rather, it is also a result of psychological overload shaped by heuristic processing and diminished cognitive ability.

Simultaneously, emotions also influence judgments and choices. Privacy concern is defined as individuals’ worries and anxieties regarding the potential misuse, unauthorized access, or secondary use of their personal information (Wang J. et al., 2025). Privacy concerns reflect the anxiety and apprehension stemming from potential privacy invasions, diminishing the disclosure intention by heightening risk perceptions. Theoretically, it align with the loss aversion principle in behavioral decision theory, which posits that individuals are more motivated to avoid potential losses than to pursue equivalent gains. Empirical evidence shows that higher levels of privacy concern negatively impact users’ willingness to disclose information (Kim et al., 2023). In the digital context, users fear potential losses, such as unauthorized access to personal data or identity theft, which heightens their perceived risks and reduces their willingness to disclose information online (Van der Schyff and Flowerday, 2023). Privacy concerns may translate into restrictive disclosure intentions, as the psychological worry of potential losses outweighs the perceived benefits of online disclosure.

Drawing on insights from behavioral decision theory, we employ three core concepts of behavioral decision theory as the key variables of this study, namely, privacy invasion experiences as the anchor in the anchoring effect, privacy fatigue as the cognitive bias caused by heuristics, and privacy concern as the emotional response of loss aversion, to understand the multi-layered disclosure decision-making process users undergo when confronting privacy issues on social media platforms.

### 2.3 Trust in social media

In digital environments, trust is often conceptualized as the belief in the integrity, competence, and benevolence of a platform or service provider (Soleimani, 2022). In the current study, we focus on trust in social media platforms. Trust in social media refers to users’ belief that the platform will act in their interest, particularly by safeguarding personal information, ensuring system reliability, and enabling secure interactions (Bansal et al., 2015). When users perceive that a social media platform is competent, ethical, and benevolent in its data handling practices, they are more likely to engage with it and disclose personal information. This trust is built upon users’ assessments of a platform’s past performance and reliability, as well as its perceived commitment to user welfare (Xie et al., 2024).

Drawing on trust theory, this study introduces trust as a central explanatory mechanism. On one hand, it explores how users’ privacy invasion experiences, privacy fatigue, and privacy concerns affect their trust in social media. Prior studies indicated that trust in social media is impacted by multiple factors (Balapour et al., 2020; Bansal et al., 2016; Krishna et al., 2023). On the other hand, it examines how such trust, in turn, influences their intention to disclose on social media platforms. In addition, trust often acts as a mediator between users’ privacy concerns and their willingness to disclose personal information (Rodríguez-Priego et al., 2023; Zhang, 2024). Even when users have strong privacy concerns, a high level of trust in a platform may encourage online disclosure, if they perceive the benefits of disclosure to outweigh the risks.

In summary, this study integrates behavioral decision theory and trust theory, offering a robust framework for understanding social media disclosure intention. Our model operationalizes privacy invasion experiences as an anchor that captures the historical context of users’ encounters with privacy invasions, influencing both their cognitive and emotional responses. Privacy fatigue, as a cognitive bias factor, represents the overload from frequent privacy invasions, which may affect users’ trust and disclosure in social media. Privacy concerns, reflecting the emotional reaction to potential breaches, are supposed to impact trust and disclosure intention. Drawing on trust theory, trust in social media embodies users’ belief in the platform’s good intention, mediating the relationship between the above privacy-related factors and the disclosure intention on social media.

## 3 Hypotheses development

Trust has been demonstrated as a crucial determinant in fostering online disclosure behaviors. Several empirical studies posit that users’ willingness to engage in online platforms is fundamentally based on their perceptions of a platform’s integrity, ability, and benevolence. For instance, Hollebeek and Macky (2019) showed that higher trust in an online environment significantly enhances consumers’ readiness to engage in transactions and share sensitive information. Similarly, research by Chen et al. (2017) and Wang et al. (2014) confirmed that trust encourages more open and extensive disclosure on digital platforms. Further, studies in the context of social media have demonstrated that when users perceive a platform as reliable, their confidence in disclosing personal data increases, which in turn, leads to higher levels of intention to disclose (Chan and Ma, 2013; Nemec Zlatolas et al., 2019).

Given the role of trust in enhancing user online engagement, we follow that trust in social media is likely to exert a positive influence on social media disclosure intention. Therefore, we supposed the following hypothesis:

*H1*: Trust in social media is positively associated with social media disclosure intention.

The role of privacy invasion experiences has been increasingly mentioned by scholars in recent years (Chen et al., 2023; Masur and Trepte, 2021) as a negative factor for online engagement. Prior research has demonstrated that privacy invasion experiences tend to heighten users’ perceptions of risk and vulnerability and significantly erode users’ trust (Hong et al., 2021; Wang et al., 2019), thereby reducing their willingness to disclose online. For instance, studies have shown that individuals who have been subjected to privacy invasions exhibit increased wariness in subsequent online interactions, resulting in lower disclosure intentions (Masur and Trepte, 2021). The negative effect is attributed to the lasting impression these experiences leave, which reinforces protective behavior and inhibits online disclosure intention. Based on these findings, we hypothesized that:

*H2*: Privacy invasion experiences are negatively associated with social media disclosure intention.

*H3*: Privacy invasion experiences are negatively associated with trust in social media.

Privacy fatigue is also receiving gradual attention. Privacy fatigue arises from the continuous cognitive and psychological burden of managing online privacy. As users are constantly exposed to privacy-related concerns and required to take protective measures, they may become overwhelmed and tired of privacy protection, leading to a sense of helplessness and disengagement. This fatigue may result in diminished trust in social media platforms, as users feel that their efforts to safeguard their privacy, including those of social media platforms, are in vain. Research shows that privacy fatigue can cause users to disengage from online engagement (Tang et al., 2021). As a result, individuals experiencing privacy fatigue may reduce their trust and reliance on social media, or even give up disclosing personal information altogether. Prior studies have suggested that privacy fatigue can lead to skepticism toward online platforms and a decline in disclosure (Shao et al., 2022). Given this, our study hypothesized that:

*H4*: Privacy fatigue is negatively associated with social media disclosure intention.

*H5*: Privacy fatigue is negatively associated with trust in social media.

Privacy concerns include emotional concerns about the possible negative consequences of disclosing online (Park et al., 2021). Empirical evidence shows that the more serious the privacy concerns, the less trust people have in online platforms (Tseng et al., 2022) and the lower their propensity to disclose personal information (Lutz et al., 2018). Users who are deeply concerned about the misuse of their personal data are more likely to limit the information they disclose because the perceived risks outweigh the potential benefits, which also stem from their trust perception. Additionally, studies (Bansal and Warkentin, 2021; Bansal et al., 2016; Xu et al., 2011) highlight that privacy concerns are a significant barrier to disclosure in online contexts. Therefore, our study hypothesized that:

*H6*: Privacy concerns are negatively associated with social media disclosure intention.

*H7*: Privacy concerns are negatively associated with trust in social media.

Prior studies have found that when individuals encounter privacy invasions, such as unauthorized access or misuse of personal data, these negative events act as powerful reminders of the risks inherent in online environments (Huang and Leu, 2020). Frequent privacy invasions overwhelm users as they navigate a complex privacy environment, leaving them with a profound sense of powerlessness. Research indicated that when social media users are frequently exposed to unauthorized access or misuse of their personal information, they may become exhausted from the constant need to protect their privacy (Van Der Schyff et al., 2023). These negative experiences induce a state of fatigue, where social media users cannot keep up with evolving online privacy challenges and feel increasingly unable to manage their privacy.

In addition, negative privacy experiences tend to amplify users’ concerns regarding the security of their personal information (Masur and Trepte, 2021). When social media users witness or experience privacy invasions, they may be more vigilant and develop stronger apprehensions about potential future breaches. This heightened state of awareness and vulnerability reinforces users’ belief that personal data may be at risk, thereby intensifying privacy concerns. Empirical studies have demonstrated that individuals with a history of privacy invasions have significantly higher levels of concern about data misuse (Yang and Liu, 2013). Based on these observations and supporting evidence, we supposed that:

*H8*: Privacy invasion experiences are positively associated with privacy fatigue in social media context.

*H9*: Privacy invasion experiences are positively associated with privacy concerns in social media context.

Trust in social media plays a dual role in this process. On the one hand, it directly improves users’ judgment of the benevolence of social media and thus increases users’ disclosure intention. On the other hand, it serves as a mediator between antecedents and disclosure intention (Lwin et al., 2016; Zhang, 2024). By fostering a sense of security, trust in social media enables users to overcome the hesitation that stems from potential privacy invasions (Zhang, 2024). Here, we proposed the mediating role of trust in the relationships between privacy invasion experiences, privacy fatigue, privacy concerns, and social media disclosure intention.

Prior studies have shown that users who have encountered privacy invasions tend to develop a sense of vulnerability, leading to decreased trust in the platform’s ability to safeguard their data (Chen et al., 2023). Reduced trust, in turn, discourages users from engaging in online disclosure, as they perceive a higher risk associated with sharing personal information online. Research in online disclosure has demonstrated that trust serves as a key factor in mitigating the negative effects of past privacy invasions (Bansal et al., 2016), as users who maintain high level of trust in online platform are more likely to continue disclosing. Additionally, as individuals become overwhelmed by constant privacy threats and the perceived burden of managing their privacy, they may develop cynicism and reduced confidence in the platform’s ability to protect their information (Shao et al., 2022). This erosion of trust subsequently lowers their willingness to disclose personal information as they perceive higher risks associated with sharing. That is to say, when trust in platform is diminished due to privacy fatigue, users are less likely to engage in online disclosure. Similarly, in the absence of trust, heightened privacy concerns lead to a greater reluctance to disclose online. Conversely, when users trust a social media platform, they may feel more comfortable sharing information despite privacy concerns. Trust functions as a psychological buffer, alleviating privacy-related anxieties and fostering disclosure behaviors (Rodríguez-Priego et al., 2023). Therefore, we hypothesized that trust in social media may serve as a mediating factor in these relationships:

*H10a*: Trust in social media mediates the relationship between privacy invasion experiences and social media disclosure intention.

*H10b*: Trust in social media mediates the relationship between privacy fatigue and social media disclosure intention.

*H10c*: Trust in social media mediates the relationship between privacy concerns and social media disclosure intention.

To ensure that the main findings are not biased by demographic differences, age and gender are included as control variables given their influence on social media disclosure intention. Prior research has demonstrated that younger individuals are generally more willing to engage in self-disclosure, while older users tend to adopt a more cautious stance (Christofides et al., 2012). Similarly, gender differences in privacy concerns and disclosure practices are well documented, with men and women exhibiting distinct patterns in both the scope and sensitivity of information disclosed (Tifferet, 2019). Therefore, controlling for age and gender helps isolate the true effects of the key variables and enhances the internal validity of the model.

**Figure 1:**
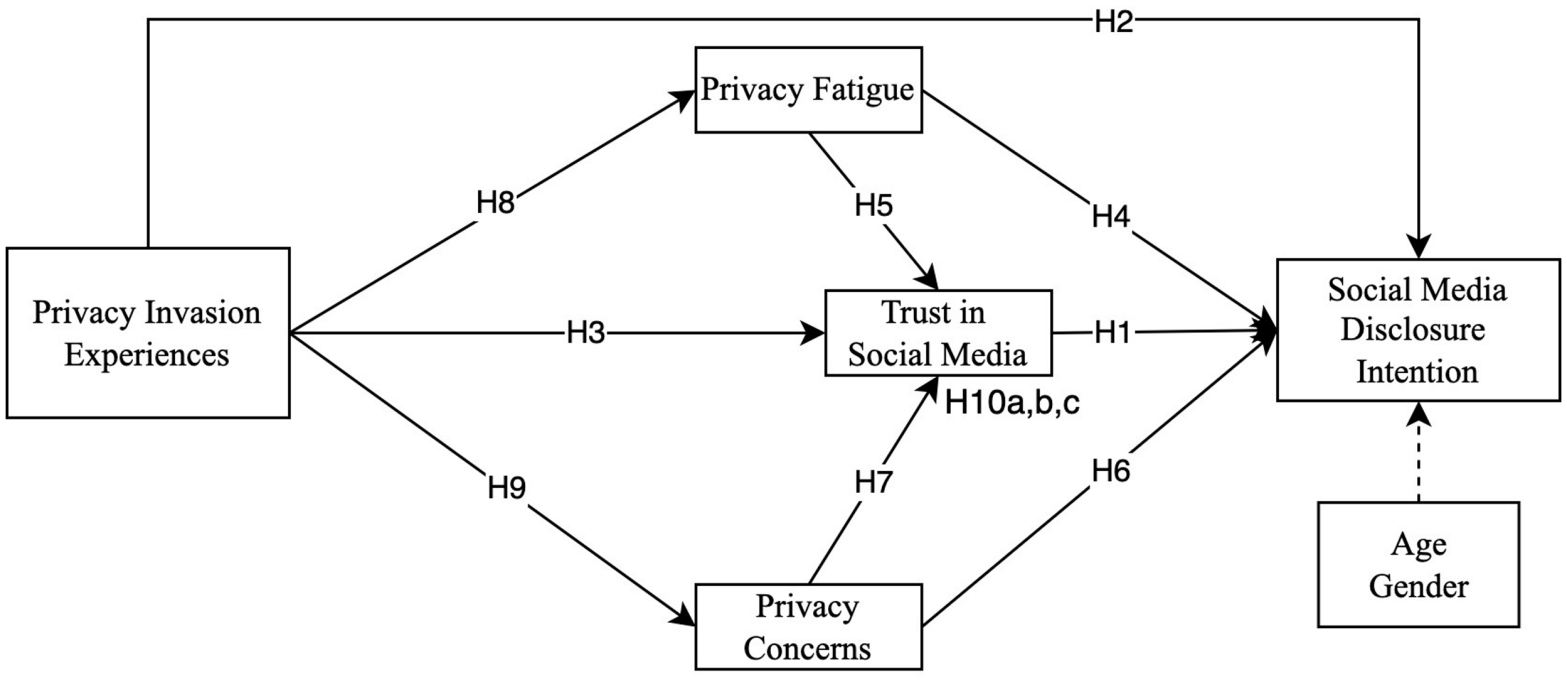
Theoretical model.

Figure 1 shows the theoretical model of this study, which integrates behavioral decision theory and trust theory to explain how privacy invasion experiences, privacy fatigue, and privacy concerns influence users’ social media disclosure intention, with trust in social media serving as a mediating role.

## 4 Methods

### 4.1 Procedures and participants

This study utilized an online survey distributed via WJX.com, and employed a network sampling technique. To ensure the reliability and clarity of the questionnaire, a pilot test was conducted prior to formal data collection. Participation in the survey was entirely voluntary and anonymous. All respondents were informed about the academic purpose of the study and assured of data confidentiality. They retained the right to withdraw from the survey at any time. The data collection was carried out in mainland China from June to December 2024. The target population consisted of Chinese social media users aged 20 to 59 years. Adolescents and older adults were excluded from the sample due to their potentially distinct usage patterns of social media (Lei et al., 2024).

This study applied the network sampling technique, a method increasingly adopted in studies focusing on interconnected digital communities (Baltar and Brunet, 2012). In this approach, initial respondents were encouraged to disseminate the survey link within their own social networks. To address potential sampling bias due to homophily in network sampling (Heckathorn and Cameron, 2017), we used demographic benchmarks from the CNNIC (2024) to monitor and guide the sampling process. Key demographic indicators such as age, gender, and residence were continuously assessed. If discrepancies in sample composition emerged, particularly with regard to underrepresented age or gender groups, targeted data supplementation was implemented to improve representativeness.

Our study initially collected a total of 1,000 responses. After removing responses with unusually short completion times, duplicate IP addresses, and highly uniform answer patterns (i.e., over 70% identical responses), 787 valid responses remained for analysis. Descriptive statistics of the final sample are presented in Table 1. The majority of participants were under the age of 39, with the 30–39 age group comprising the largest proportion (37.4%). The gender distribution was balanced (52.7% male and 47.3% female). In terms of residence, 44.9% of respondents lived in provincial capital cities. Additionally, 36.6% of participants reported spending more than 5 h per day on social media. The sample is broadly representative of Chinese social media users.

**Table 1:** Profile of respondents (*n* = 787).

Characteristics	Frequency	Percentage
Age		
20–29	208	26.4%
30–39	294	37.4%
40–49	193	24.5%
50–59	92	11.7%
Gender		
Male	415	52.7%
Female	372	47.3%
Residence		
Rural area	44	5.6%
County	83	10.5%
Ordinary city	307	39.0%
Provincial capital	353	44.9%
Social media daily usage time		
Less than 1 h	58	7.4%
1–3 h	209	26.6%
3–5 h	232	29.5%
More than 5 h	288	36.6%

### 4.2 Measures

Our study includes five constructs, namely privacy invasion experiences, privacy fatigue, privacy concerns, trust in social media, and social media disclosure intention. We adapted the measurement items for these constructs from well-established scales in prior literature and localized them to fit our specific research context. Table 2 shows the operationalized definition of constructs. We adapted the items of privacy invasion experiences in Masur and Trepte (2021) to fit the research topic of this study. The measurement items for privacy fatigue were adapted from Choi et al. (2018), with modifications to align with the social media environment. Privacy concerns were measured using an adapted version of the scale proposed by Xu et al. (2008), ensuring its relevance to users’ privacy perceptions on social media platforms. The trust in social media construct was derived from Lo and Cindy (2010), incorporating elements that reflect trust in the context of social media interactions. Lastly, social media disclosure intention was measured using items from Zhu et al. (2022) and Fan et al. (2021), assessing users’ willingness to share personal information on social media. The measurement items of constructs are shown in Appendix A. All constructs were measured using a five-point Likert scale ranging from “strongly disagree” (1) to “strongly agree” (5). To ensure the validity and reliability of the measurement instruments, these indicators were validated by an expert panel. Additionally, a pilot test was conducted with a sample of social media users to evaluate the measurement items in advance. The pilot test results confirmed that all constructs exhibited good internal consistency and convergent validity, supporting their use for further empirical analysis.

**Table 2:** Operationalization of constructs.

Constructs	Constructs definition	Source
Social Media Disclosure Intention	Individuals’ behavioral tendency to reveal personal information on social media platforms.	Bansal et al. (2010)
Trust in social media	Individuals’ belief that social media platforms will act in their interest by protecting their data, ensuring reliable system functioning, and enabling secure interactions.	Bansal et al. (2015)
Privacy Fatigue	Individuals’ psychological state of weariness and resignation that arises when they feel overwhelmed by the effort required to manage and protect their personal data on social media.	Chen et al. (2023)
Privacy Concerns	Social media users’ worries and anxieties regarding the potential misuse, unauthorized access, or secondary use of their personal information.	Wang J. et al. (2025)
Privacy Invasion Experiences	Individuals’ past encounters with unauthorized access, misuse, or exposure of their personal information on social media environments.	Huang and Leu (2020)

In addition, to assess potential common method variance, a variable unrelated to this study, namely Neighborhood Aesthetics (Carlson et al., 2012), was incorporated as a marker variable. This construct consists of four items and is also measured using a five-point Likert scale. These items were presented alongside the main variables within the questionnaire.

### 4.3 Common method variance

To minimize potential common method variance (CMV) due to data collected from a single questionnaire, this study adopted both procedural design and *post hoc* detection. At the design stage, we adopted a variety of techniques, including ensuring anonymity and reducing item ambiguity, and shuffling the order of questions. In post hoc, two statistical approaches were employed to assess the presence of CMV, Harman’s single-factor test and the marker variable technique. Firstly, we conducted Harman’s single-factor test. We performed an unrotated principal component analysis in SPSS, including all items from the measurement scales. The results indicated that the first factor load of an interpretable variable accounted for only 29.5% of the total variance, which is well below the 50% threshold recommended by Eichhorn (2003), suggesting that CMV is not a significant concern.

Since Harman’s test is considered a relatively weak diagnostic tool for CMV (Fuller et al., 2016), we further applied the PLS marker variable technique, as suggested by Chin et al. (2013) and Simmering et al. (2015). Regarding the selection of the marker variable, prior research suggests that as few as four items can effectively detect and control over 70% of CMV (Chin et al., 2013). We selected “Neighborhood Aesthetics” from Carlson et al. (2012) as the marker variable since it comprises four items and has no conceptual or theoretical relationship with any of the variables in our study. The marker variable was incorporated into the baseline model and linked to all observed variables as a latent method factor. We then used SmartPLS 4 to compare changes in R^2^ values and path coefficients between the baseline model and marker model (including the marker construct). As shown in Tables 3, 4, the introduction of the marker variable resulted in changes of less than 10% in path coefficients and less than 0.2 in R^2^, consistent with the threshold suggested by Chin et al. (2013). These findings confirm that CMV does not pose a substantial threat in this study.

**Table 3:** Path coefficients changes between baseline and marker models.

Relationships	Path coefficients		Change
	Baseline model	Marker model	
Privacy Concerns - > Social Media Disclosure Intention	−0.261	−0.257	−1.5%
Privacy Concerns - > Trust in Social Media	−0.158	−0.160	1.3%
Privacy Fatigue - > Social Media Disclosure Intention	−0.138	−0.140	1.4%
Privacy Fatigue - > Trust in Social Media	−0.083	−0.080	−3.6%
Privacy Invasion Experience - > Privacy Concerns	0.287	0.286	−0.3%
Privacy Invasion Experience - > Privacy Fatigue	0.287	0.287	0.0%
Privacy Invasion Experience - > Social Media Disclosure Intention	−0.085	−0.086	1.2%
Privacy Invasion Experience - > Trust in Social Media	−0.126	−0.126	0.0%
Trust in Social Media - > Social Media Disclosure Intention	0.323	0.324	0.3%

**Table 4:** *R*^2^ changes between baseline and marker models.

Constructs	*R* ^2^		Change
	Baseline model	Marker model	
Privacy Concerns	0.082	0.085	0.003
Privacy Fatigue	0.082	0.083	0.001
Social Media Disclosure Intention	0.325	0.326	0.001
Trust in Social Media	0.079	0.081	0.002

## 5 Data analysis

This study employed the PLS-SEM approach to test the research model, following the two-stage analysis method recommended by Hair et al. (2021), including measurement model assessment and structural model assessment.

### 5.1 The measurement model assessment

The measurement model assessment demonstrated satisfactory reliability and validity. The Cronbach’s *α* (CA) for all constructs ranged from 0.743 to 0.846, while the composite reliability (CR) values ranged from 0.854 to 0.890, indicating strong internal consistency and reliability (Hair et al., 2021). Additionally, the outer loadings of all indicators exceeded the recommended threshold of 0.7, confirming adequate indicator reliability. The AVE for all constructs was above the threshold of 0.5, with values ranging from 0.588 to 0.664, suggesting satisfactory convergent validity (Fornell and Larcker, 1981). The results are presented in Table 5. To assess discriminant validity, the Heterotrait-Monotrait (HTMT) ratio technique was applied (Henseler et al., 2015). As shown in Table 6, all HTMT values were below the critical threshold of 0.85, confirming that discriminant validity was established.

**Table 5:** Results of measurement model test.

Constructs	Items	Outer Loading	Cronbach’s α	rho_A	CR	AVE
Privacy invasion experiences	PIE1	0.791	0.743	0.744	0.854	0.661
	PIE2	0.818				
	PIE3	0.829				
Privacy fatigue	PF1	0.794	0.832	0.833	0.888	0.664
	PF2	0.815				
	PF3	0.817				
	PF4	0.833				
Privacy concerns	PC1	0.736	0.825	0.829	0.877	0.588
	PC2	0.743				
	PC3	0.763				
	PC4	0.795				
	PC5	0.797				
Trust in social media	TSM1	0.780	0.846	0.849	0.890	0.619
	TSM2	0.792				
	TSM3	0.757				
	TSM4	0.791				
	TSM5	0.814				
Social media disclosure intention	SMDI1	0.789	0.837	0.840	0.884	0.605
	SMDI2	0.830				
	SMDI3	0.782				
	SMDI4	0.755				
	SMDI5	0.730				

**Table 6:** Discriminant validity Heterotrait-Monotrait (HTMT).

Constructs	1	2	3	4	5
1. Privacy Concerns					
2. Privacy Fatigue	0.630				
3. Privacy Invasion Experience	0.365	0.364			
4. Social Media Disclosure Intention	0.521	0.433	0.333		
5. Trust in Social Media	0.278	0.240	0.245	0.506	

### 5.2 The structural model assessment

After establishing the reliability and validity of the research instrument, we employed a bootstrapping procedure with 5,000 resamples using the one-tailed test to estimate the significance of the path coefficients (Hair et al., 2021). First, we assessed the VIF of the structural model, as path coefficients between constructs are calculated based on regression analysis. This step ensures that multicollinearity does not distort the regression results. The VIF values of all predictors did not exceed the recommended threshold of 3.3 (Hair et al., 2021), indicating that multicollinearity was not a concern (see Table 7). Next, we evaluated the structural model using key statistical indicators, including path coefficients (*β*), *t*-values, *p*-value, confidence interval, effect size (*f*^2^), predictive relevance (*Q*^2^), and the coefficient of determination (*R*^2^). The results provide insights into the relationships between constructs and the explanatory power of the structural model.

**Table 7:** Results summary of the structural model assessment.

Code	Relationships	Path coefficients	Standard deviation	T-value	P-value	95%BCa CI		*f^2^*	VIF	Decision
						LB	UB			
H1	Trust in Social Media - > Social Media Disclosure Intention	0.313	0.036	8.662	0.000	0.240	0.381	0.134	1.097	Accepted
H2	Privacy Invasion Experience - > Social Media Disclosure Intention	−0.079	0.039	2.011	0.044	−0.152	0.001	0.008	1.144	Accepted
H3	Privacy Invasion Experience - > Trust in Social Media	−0.126	0.037	3.410	0.001	−0.196	−0.053	0.015	1.121	Accepted
H4	Privacy Fatigue - > Social Media Disclosure Intention	−0.133	0.038	3.473	0.001	−0.207	−0.057	0.018	1.427	Accepted
H5	Privacy Fatigue - > Trust in Social Media	−0.083	0.042	1.957	0.050	−0.164	0.002	0.005	1.411	Rejected
H6	Privacy Concerns - > Social Media Disclosure Intention	−0.257	0.034	7.651	0.000	−0.319	−0.187	0.069	1.442	Accepted
H7	Privacy Concerns - > Trust in Social Media	−0.158	0.038	4.167	0.000	−0.230	−0.081	0.019	1.411	Accepted
H8	Privacy Invasion Experience - > Privacy Fatigue	0.287	0.038	7.551	0.000	0.212	0.359	0.090	1.000	Accepted
H9	Privacy Invasion Experience - > Privacy Concerns	0.287	0.037	7.800	0.000	0.209	0.353	0.090	1.000	Accepted
H10a	Privacy Invasion Experience - > Trust in Social Media - > Social Media Disclosure Intention	−0.039	0.013	3.116	0.002	−0.066	−0.017	–	–	Accepted
H10b	Privacy Fatigue - > Trust in Social Media - > Social Media Disclosure Intention	−0.026	0.014	1.865	0.062	−0.054	0.000	–	–	Rejected
H10c	Privacy Concerns - > Trust in Social Media - > Social Media Disclosure Intention	−0.049	0.013	3.808	0.000	−0.077	−0.025	–	–	Accepted
	Age - > Social Media Disclosure Intention	0.005	0.031	0.163	0.871	−0.056	0.064	–	–	–
	Gender - > Social Media Disclosure Intention	0.194	0.061	3.210	0.001	0.071	0.311	–	–	–

## 6 Results

**Figure 2:**
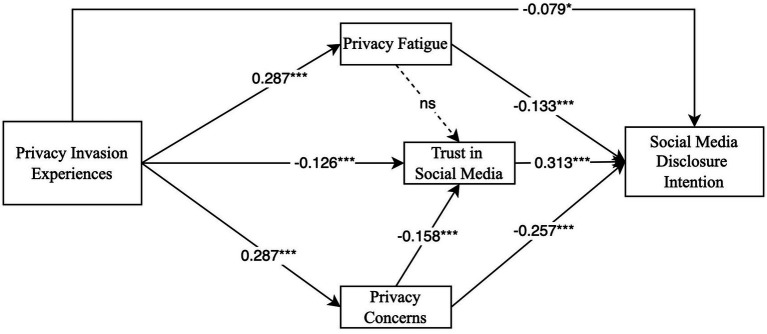
The structural model test results.

Concerning direct effect, the results confirmed 8 hypotheses and rejected 1 hypothesis (see Figure 2). Specifically, the findings indicated that trust in social media has a significant positive association with social media disclosure intention (*β* = 0.313, *t* = 8.662, *p* < 0.001), supporting H1. This indicated that users are more willing to disclose personal information when they perceive the platform as trustworthy. Additionally, privacy invasion experiences (*β* = −0.079, *t* = 2.011, *p* = 0.044), privacy fatigue (*β* = −0.133, *t* = 3.473, *p* < 0.001), and privacy concerns (*β* = −0.257, *t* = 7.651, *p* < 0.001) negatively influence social media disclosure intention, confirming H2, H4 and H6. Moreover, privacy invasion experiences is negatively associated with trust in social media (*β* = −0.126, *t* = 3.410, *p* = 0.001), supporting H3, and privacy concerns also have a negative influence on trust in social media (*β* = −0.158, *t* = 4.167, *p* < 0.001), supporting H7. This echoes findings from prior literature that privacy invasion experiences and privacy concerns erode trust in digital platforms (Bansal et al., 2016). Furthermore, privacy invasion experiences increase both privacy fatigue (*β* = 0.287, *t* = 7.551, *p* < 0.001) and privacy concerns (*β* = 2.287, *t* = 7.800, *p* < 0.001), supporting H8 and H9, which suggest that users who experience privacy invasions become more fatigued and concerned as these experience increases.

Converse to expectation, H5 was found to be non-significant. Privacy fatigue does not significantly reduce trust in social media (*β* = −0.083, *t* = 1.957, *p* = 0.050), implying that privacy fatigue caused by complex operations may not directly lead to users losing trust in social media.

As for indirect effect, this study revealed that trust in social media mediates the negative relationship between privacy invasion experiences and social media disclosure intention (*β* = −0.039, *t* = 3.116, *p* = 0.002), as well as between privacy concerns and disclosure intention (*β* = −0.049, *t* = 3.808, *p* < 0.001), confirming H10a and H10c. These findings suggested that users who experience privacy invasions or hold strong privacy concerns tend to disclose less, partly through their diminished trust in social media. This is consistent with previous studies indicating that platform-trust acts as a crucial mediator in privacy-related behaviors (Lwin et al., 2016; Rodríguez-Priego et al., 2023; Zhang, 2024). However, the mediating role of social media trust in the relationship between privacy fatigue and social media disclosure intention was not significant (*β* = −0.026, *t* = 1.865, *p* = 0.054), suggesting H10b was rejected. This distinction highlights the nuanced role of different privacy-related constructs and suggests that not all forms of privacy-related stress translate into diminished trust in social media.

We further examined the effects of control variables. Age did not significantly associate with social media disclosure intention, which may be attributed to the narrowing digital divide in China. With over two decades of widespread social media use, adults in 50s tend to exhibit similar levels of digital familiarity and privacy-related behavior to those in 20s. In contrast, gender showed a significant association. Male users reported higher disclosure intention than female, likely because females are more sensitive to privacy risks (Malik et al., 2016) and thus more cautious on social media (Tifferet, 2019).

Following hypothesis testing, we evaluated the effect size (*f*^2^) to determine the practical significance of the relationships in the model. According to Cohen (1988), thresholds of 0.02, 0.15, and 0.35 represent small, medium, and large effect sizes of direct effects, respectively. The results indicate that the effect sizes in our study fall within the small effect range. Then, we evaluated the coefficient of determination (*R*^2^) to measure the model’s predictive accuracy. Based on the criteria of Chin (1998), *R*^2^ values of 0.19, 0.33, and 0.67 are classified as weak, moderate, and substantial levels of explanatory power, respectively. The *R*^2^ value for social media disclosure intention in our study is 0.325, indicating that the model explains about 32.5% of the variance in users’ social media disclosure intention, which is considered moderate. To assess model robustness, we assessed the predictive relevance (*Q*^2^) using the cross-validated redundancy measure (Hair et al., 2021). The results in Table 8 show that Q^2^ values are all above 0 (from 0.047 to 0.191), confirming that our model possesses good predictive relevance and robustness. Furthermore, we conduct the sensitivity analyses by removing the control variables from the baseline model and comparing the results. The key indicators, including path coefficients, their significance levels, and R^2^ values, remained largely unchanged, indicating model stability. Additionally, comparisons between the baseline model and the marker model also revealed negligible differences in structural assessment, which further enhanced the statistical robustness of the study.

**Table 8:** Results of *R*^2^ and *Q*^2^.

Constructs	*R* ^2^	*Q* ^2^
Privacy Concerns	0.082	0.047
Privacy Fatigue	0.082	0.054
Trust in Social Media	0.079	0.047
Social Media Disclosure Intention	0.334	0.191

### 6.1 Importance-performance map analysis

**Figure 3:**
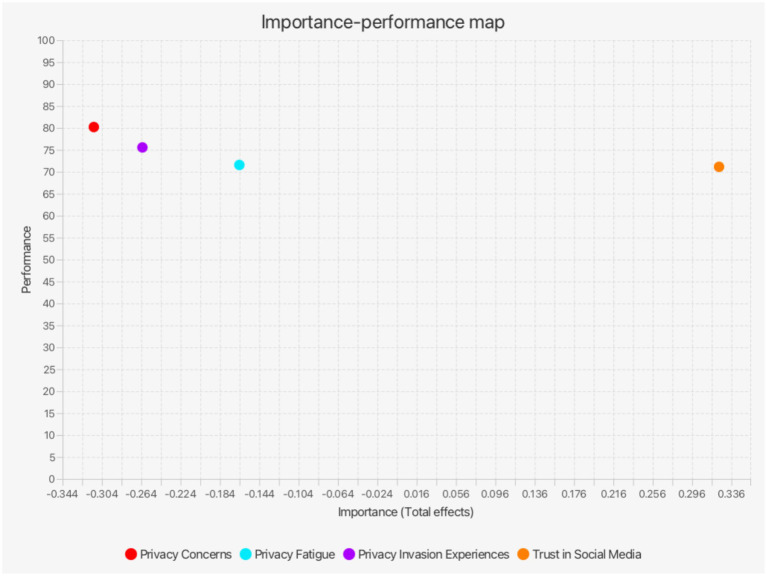
The importance-performance map of social media disclosure intention.

The Importance-Performance Map Analysis (IPMA) provides insights beyond traditional path coefficient analysis by considering both importance (total effects) and performance (average latent variable scores). It was shown to be of great use in explaining which factors have the greatest impact on the target construct and which improvements would be most effective (Streukens et al., 2017). We conducted IPMA to further investigate whether the positive effect of trust in social media on disclosure intention outweighs the negative effects of privacy-related factors (privacy invasion experiences, privacy fatigue, and privacy concerns). Additionally, this analysis helps to determine which of the negative factors exert the greatest influence on social media disclosure intention (Figure 3).

Table 9 reveals that trust in social media is the most influential positive factor (importance = 0.323), surpassing all negative factors. This reinforces its role as a key driver in users’ online behavior. Privacy concerns exert the strongest negative impact (importance = −0.312), indicating that users who perceive high privacy risks are significantly less likely to disclose personal information. Furthermore, privacy fatigue exhibits the lowest importance, suggesting that while some users feel overwhelmed or resigned regarding privacy risks, this fatigue alone does not substantially deter disclosure compared to other factors. Although trust in social media demonstrates the highest importance, it records the lowest performance score (performance = 71.146), indicating that users currently exhibit a relatively low level of trust in these platforms. In other words, cultivating users’ trust is not only critical but also presently underdeveloped. The gap between the high importance and low performance of trust in social media underscores that enhancing users’ trust could substantially improve their intention to disclose on social media. This finding offers practical insights for social media platforms that strengthening users’ trust may prove to be a more effective strategy than merely addressing negative factors such as privacy fatigue or privacy concerns.

**Table 9:** Results of importance-performance map analysis (IPMA).

Constructs	Importance (total effects)	Performance
Privacy Concerns	−0.312	80.213
Privacy Fatigue	−0.164	71.567
Privacy Invasion Experiences	−0.263	75.565
Trust in Social Media	0.323	71.146

## 7 Discussion, implications, limitations and conclusion

The purpose of this study is to explore the relationship among privacy invasion experiences, privacy fatigue, privacy concerns, trust in social media and social media disclosure intention by integrating behavioral decision theory and trust theory into a comprehensive model. The results confirm that privacy invasion experiences, privacy fatigue, privacy concerns are negatively associated with social media disclosure intention, while trust in social media is the most important factors in influencing social media disclosure intention. In addition, trust in social media mediates the relationships between privacy invasion experiences and privacy concerns, and social media disclosure intention. This findings help to clarify how individuals’ past experiences with privacy invasion, their cognitive evaluations of privacy risks, and emotional reactions such as concern influence their trust in social media, which in turn collectively shape their intention to disclose personal information on social media platforms.

We found that trust in social media positively influences disclosure, supporting previous studies suggesting that trust is a key enabler of online information disclosure (Chen et al., 2017; Rodríguez-Priego et al., 2023; Soleimani, 2022; Wang et al., 2014; Zhang, 2024). Our results further extend these findings by demonstrating that trust in social media has the strongest importance among all examined factors, outweighing privacy-related behavioral decision factors. However, the IPMA results indicate that the negative impact of privacy concerns is nearly as strong as the positive influence of trust. This raises an theoretical question, can trust in social media “offset” privacy concerns? While some studies argue that trust mitigates privacy concerns and encourages information disclosure, e.g., Cherif et al. (2021), we indicate that, rather than directly eliminating privacy concerns, social media trust operates primarily at a psychological level by offering emotional reassurance, platform credibility, and affective comfort. In contrast, privacy concerns stem from rational cognitive evaluations of risk and control. From this perspective, trust and privacy concerns are not merely opposing forces on disclosure intention but instead function as parallel constructs that influence disclosure from different dimensions. In other words, even when users exhibit a high level of trust, it may not fully neutralize the adverse effects of privacy concerns. Therefore, although enhancing trust is crucial, it alone is insufficient. Social media platforms must also address users’ privacy-related worries through concrete measures to effectively promote disclosure behavior.

Among the privacy-related factors, privacy concerns exhibited the most substantial negative influence on disclosure intention, which is consistent with previous findings (Ha et al., 2021; Tang et al., 2021). This result reinforces the notion that users who perceive greater privacy concerns are less willing to disclose personal information. Additionally, we found privacy invasion experiences significantly decreased disclosure intention on social media. This finding consists of some prior studies that argue past negative experiences directly reduce disclosure (Smith et al., 2011; Xu et al., 2014). Moreover, our study highlights the role of privacy fatigue as a distinct construct influencing disclosure. While previous research has examined privacy fatigue as a consequence of frequent privacy invasions (Choi et al., 2018), its effect on trust and disclosure remains understudied. Our results show that privacy fatigue has a negative impact on disclosure intention, but the impact on trust is not significant. This suggests that while users may reduce their disclosure intentions due to complex privacy operations and resulting fatigue, this reduction is not related to trust. This non-significant relationship may be explained by emotional exhaustion and user apathy. Users overwhelmed by complex privacy management and continuous exposure to privacy risks may become apathetic. This sense of resignation may weaken the link between privacy fatigue and trust in social media. Future studies could further investigate this path using psychological constructs such as user apathy or privacy resignation.

Furthermore, our findings indicate that trust in social media significantly mediates the relationships between privacy concerns, privacy invasion experiences, and social media disclosure intention, further demonstrating the strong mediating role of trust. Although privacy concerns directly reduce users’ willingness to disclose, this effect often occurs through diminished trust in social media. This aligns with prior research suggesting that trust is a key mechanism through which users’ privacy-related concerns influence disclosure behaviors (e.g., Bansal et al., 2016; Wang J. et al., 2025). However, the mediating effect of trust is not observed in the relationship between privacy fatigue and disclosure intention. A possible explanation is that privacy fatigue, as a cognitive and emotional exhaustion stemming from prolonged exposure to complex and repetitive privacy threats, may lead users to reduce self-disclosure independently of their trust evaluations. In other words, users who experience privacy fatigue may not necessarily distrust the platform, but rather feel overwhelmed toward managing their privacy, which leads to disengagement in disclosure behaviors. This perspective is consistent with the argument that privacy fatigue reflects withdrawal rather than active distrust (Lyu et al., 2024; Tang et al., 2021). Therefore, while trust serves as a key mediator in certain privacy-related paths, its role in the context of fatigue-induced disclosure reduction appears to be limited.

### 7.1 Theoretical implications

This study advances the theoretical development of social media privacy research by integrating behavioral decision theory and trust theory into the context of social media disclosure. While prior research has predominantly conceptualized disclosure decisions as rational cost–benefit calculations, behavioral decision theory emphasizes the influence of psychological heuristics such as anchoring effects, cognitive biases, and loss aversion (Gilovich et al., 2002; Tversky and Kahneman, 1974). Drawing from this framework, this study conceptualizes privacy invasion experiences as anchoring events that serve as cognitive reference points for future disclosure decisions. Privacy fatigue is interpreted as a manifestation of cognitive overload resulting from the continual demands of privacy management, and privacy concerns are framed as expressions of loss aversion, reflecting users’ fear of potential harm from personal information disclosure.

By applying these constructs to the digital disclosure context, this study extends the application of behavioral decision theory beyond its traditional domains in economics and consumer behavior to the emerging field of digital privacy. Simultaneously, the incorporation of trust theory allows for the examination of trust as a psychological mechanism that mediates the relationship between privacy-related emotional responses and users’ disclosure intentions. Findings demonstrate that trust in social media significantly mediates the effects of privacy concerns and privacy invasion experiences on social media disclosure intention, highlighting its pivotal role as a psychological bridge between emotional perception and behavioral intention.

Using IPMA, this study compares the positive and negative factors influencing social media disclosure intention and finds that trust in social media emerges as the most influential positive predictor, surpassing all negative factors, including privacy concerns, which exert the strongest negative effect. However, we argue that the positive effect of trust do not offset the negative influence of privacy concerns. This is because these two constructs operate through distinct psychological mechanisms. Trust in social media functions as emotional reassurance rooted in perceived credibility and platform reliability, whereas privacy concerns stem from evaluations of risk and control. They influence user behavior in parallel but non-compensatory ways. This finding enriches the theoretical understanding of the trust-privacy concern framework.

Furthermore, this study contributes novel insights by introducing privacy fatigue as a predictive factor. Although prior literature has recognized privacy fatigue as a product of sustained privacy threats (Chen et al., 2023), its role in shaping trust and disclosure intention remains underexplored. By establishing privacy fatigue as a negative predictor of disclosure intention, this study sheds light on how emotional exhaustion impacts digital behaviors. In an era of increasingly complex digital privacy challenges, the findings offer a new theoretical lens to understand the psychological mechanisms linking privacy and trust to disclosure on social media.

### 7.2 Practical implications

This study offers actionable insights for platform designers, users, and policy makers aiming to foster a more secure and trust-oriented social media environment. First, the negative impacts of privacy fatigue and privacy concerns on users’ disclosure intention underscore the urgency for platforms to reduce privacy-related burdens and increase transparency in data practice. By simplifying privacy controls and minimizing users’ management load, platforms can mitigate privacy fatigue and privacy concerns to restore users’ intention to disclose on social media.

Given that trust in social media emerged as the strongest predictor of disclosure intention with a low performance, platforms must prioritize trust-building of users. Specifically, transparent data practices, verified security certifications, and robust user feedback mechanisms should be implemented to enhance users’ perceived reliability of the platform. Importantly, trust-building should extend beyond system design to encompass user-oriented communication policies. Educating users about how their data is protected, and offering personalized privacy settings, can enhance users’ trust while respecting individual comfort levels with disclosure, which should be put a premium on by policy makers and platform designers. For users, psychological interventions such as mindfulness training (Gu et al., 2022) and the improvement of online privacy literacy (Neves et al., 2024) may contribute to reducing users’ privacy fatigue and alleviating their concerns about online disclosure. As privacy challenges continue to evolve, platform strategies that address users’ emotional and psychological privacy problems will be better positioned to foster sustainable user relationships in digital environments.

### 7.3 Limitations

Although the contribution of this study is clear, it still has limitations for future research to explore. First, while this research aims to explain how individuals make disclosure decisions under privacy uncertainty, it does not account for broader social, cultural, or personality-related factors that may influence user behavior. Factors such as individual risk tolerance, cultural values, and personality traits (e.g., openness or neuroticism) may moderate users’ privacy perceptions and trust formation. Future research could integrate these factors to develop a more comprehensive understanding of privacy-related decision-making. Second, the study treats social media as a homogeneous environment, without differentiating between platform-specific characteristics. However, different platforms (e.g., TikTok, WeChat, Weibo) have varying privacy settings, affordances, and user interaction patterns, which may influence both trust and disclosure behaviors. Future research should consider platform-level variables or conduct comparative studies across different social media environments. Third, methodological limitations must be acknowledged. This study employs a cross-sectional design and network sampling, which constrain the ability to draw causal inferences and generalize findings beyond the current sample. Longitudinal or experimental designs would provide stronger evidence of causal relationships among privacy experiences, trust, and disclosure intention and allow for continued investigation of the trust-privacy concern relationship. Additionally, expanding sampling strategies to include a more diverse population could enhance the external validity of future studies. Finally, since privacy norms, risk perceptions, and trust formation are deeply influenced by cultural and regulatory environments (Wang et al., 2020), the findings may not be generalizable to other cultural contexts. Future research should adopt a cross-cultural comparative approach to explore how cultural and institutional differences influence the privacy-trust-disclosure process in different regions.

### 7.4 Conclusion

This study integrates behavioral decision theory and trust theory to examine how privacy invasion experiences, privacy fatigue, and privacy concerns influence users’ trust and disclosure intention on social media. Results from a sample of Chinese users reveal that while all three privacy-related factors negatively affect disclosure intention, trust in social media serves as the strongest positive predictor and mediates the effects of both privacy invasion experiences and privacy concerns.

By conceptualizing privacy fatigue and privacy concerns as cognitive and emotional responses, and identifying trust as a central psychological mechanism, this study expands the theoretical application of behavioral decision theory in digital privacy contexts. The inclusion of privacy fatigue offers deeper insight into how emotional exhaustion influences online disclosure behavior. These findings contribute to understanding the psychological processes underlying online disclosure and provide practical implications for designing trustworthy and privacy-sensitive platforms.

## Data Availability

The raw data supporting the conclusions of this article will be made available by the authors, without undue reservation.

## Appendix A

### The measurement items of constructs

**Table:**

Main constructs and measurement items		Sources
Privacy Invasion Experiences (PIE)		Masur and Trepte (2021)
	In my past social media usage experiences, I encountered the following issues very frequently:	
PIE1	identity theft.	
PIE2	financial fraud.	
PIE3	unnecessary requests to disclose personal information.	
Privacy Fatigue (PF)		Choi et al. (2018)
PF1	I feel emotionally drained from dealing with privacy issues in social media environment.	
PF2	I am tired of social media privacy issues.	
PF3	I have become less enthusiastic in protecting personal information provided to social media platforms.	
PF4	I doubt the significance of social media privacy issues more often.	
Privacy Concerns (PC)		Xu et al. (2008)
PC1	It usually bothers me when social media platform asks me for personal information.	
PC2	I am concerned that social media are collecting too much personal information about me.	
PC3	I am concerned that unauthorized people may access my personal information.	
PC4	I am concerned that social media may keep my personal information in a non-accurate manner.	
PC5	I am concerned about submitting information to social media platforms.	
Trust in social media (TSM)		Lo and Cindy (2010)
TSM1	I believe that social media platforms would act in my best interest when dealing with my personal information.	
TSM2	The social media platforms are interested in protecting my personal information according to the preferences I specify.	
TSM3	The social media platforms would fulfill their promises related to the personal information provided by me.	
TSM4	The social media platforms handle personal information submitted by users in a competent fashion.	
TSM5	The social media platforms perform their role of managing my personal information according to my privacy settings very well.	
Social Media Disclosure Intention (SMDI)		Zhu et al. (2022) and Fan et al. (2021)
SMDI1	I feel happy to disclose my private information on social media.	
SMDI2	I often give out my personal information on social media.	
SMDI3	I will continue to disclose my personal information on social media in the future.	
SMDI4	I will disclose more personal information on social media in the future.	
SMDI5	I often disclose real, detailed personal information on social media.	
